# Pathotypes and Simple Sequence Repeat (SSR)-Based Genetic Diversity of *Phytophthora sojae* Isolates in the Republic of Korea

**DOI:** 10.3390/microorganisms13030478

**Published:** 2025-02-21

**Authors:** Ngoc Ha Luong, In-Jeong Kang, Hee Jin You, Sungwoo Lee

**Affiliations:** 1 Department of Crop Science, Chungnam National University, Daejeon 34134, Republic of Korea; luongngocha.biotech@gmail.com (N.H.L.); heejinyou0410@gmail.com (H.J.Y.); 2 Division of Crop Cultivation and Environment Research, National Institute of Crop Science, Suwon 16613, Republic of Korea; fairjung@korea.kr

**Keywords:** soybean, *Phytophthora sojae*, pathotype, genetic diversity, differential variety

## Abstract

*Phytophthora sojae* is the causal agent of the Phytophthora root and stem rot in soybean, which has resulted in a significant increase in the incidence of the disease and substantial yield losses on a global scale. The proliferation of *Phytophthora sojae* can be mitigated through the development of Phytophthora-resistant soybean cultivars. A fundamental understanding of the genetic diversity and dynamic changes within the *P. sojae* population is essential for disease management and the development of new *P. sojae*-resistant varieties. Although a large number of pathogen samples can lead to more comprehensive interpretations and better conclusions, only six indigenous *P. sojae* isolates were available in the Republic of Korea at the time of the experiments. Due to the limited availability, this study preliminarily aimed to assess the pathotypes and genetic variation of the six *P. sojae* isolates collected in the Republic of Korea. The virulence patterns of all the six *P. sojae* isolates differed based on the 15 soybean differentials known for *P. sojae* resistance. The six isolates displayed high levels of pathotype complexities, ranging from 8 to 15, which is notably higher than those observed in other countries. Furthermore, 18 of the 21 simple sequence repeat markers used exhibited polymorphisms. The mean allele number (3.8) shows higher genetic variability compared to that (2.5) of isolates from the USA. The gene diversity (0.624) and the mean polymorphic information content (0.579) also displayed high levels of variation among the six isolates. A low mean heterozygosity (0.019) indicated a rare but possible outcrossing between the isolates, which was detected by the SSR marker PS07. Genetic dissimilarity assessments were employed to categorize the six *P. sojae* isolates into three groups using a neighbor-joining phylogenetic tree and principal component analysis. Although on a small scale, the phenotypic and genotypic assay results obtained indicated a significant variability in the pathotypes and genetic variation within the *P. sojae* isolates in the Republic of Korea. Though limited in scope, these results will be a cornerstone for elucidating the virulence pathotype and genetic diversity of the *P. sojae* population in future analyses. These findings also have the potential to improve the soybean breeding strategies aimed at enhancing resistance to *P. sojae* in the Republic of Korea.

## 1. Introduction

The soilborne plant pathogen *Phytophthora sojae* was first identified in the USA in 1948 [1,2]. It is responsible for Phytophthora root and stem rot (PRSR), a soybean disease found worldwide [1,3,4,5,6], causing an annual economic loss estimated to be between 1 and 2 billion US dollars [7,8]. *P. sojae* is recognized as a plant quarantine pathogen in several countries (available at https://gd.eppo.int/taxon/PHYTMS/categorization, last accessed on 7 February 2025) and is listed in the U.S. Regulated Plant Pest Table (available at https://www.aphis.usda.gov/plant-imports/regulated-pest-list, last accessed on 7 February 2025) of Animal and Plant Health Inspection Service, USDA, due to its potential threat to agricultural productivity and economic stability. With the increasing global trade of soybeans, the spread of *P. sojae* presents a significant risk to soybean production worldwide. This disease has been ranked as one of the topmost destructive soybean diseases over the past few decades [7,9,10,11,12]. Due to its significance, the pathotypes and genetic diversity of *P. sojae* isolates have been well documented in several countries, including the USA, Australia, Japan, Brazil, and China [13,14,15,16,17,18,19,20].

The management of PRSR mainly depends on using the *R*-gene type resistance, controlled by a single dominant resistance gene called “Resistance to *Phytophthora sojae*” (*Rps*). The *Rps* genes provide complete immune-type resistance that blocks the infection by the pathogen via a hypersensitive response of the infected tissues [1]. More than 30 *Rps* genes/alleles have been mapped to ten chromosomes [21,22]. This type of resistance generally follows the classical gene-for-gene model [23]. An *Rps* gene is effective against a limited number of *P. sojae* isolates carrying the cognate avirulence (*Avr*) gene and provides complete protection to the *P*. *sojae* isolates. The evolution or rapid shifts in the local *P. sojae* isolates often shortens the longevity of the deployed *Rps* genes, as these new isolates could neutralize the effectiveness of the *Rps*-mediated resistance [1]. Therefore, understanding the pathotypes of *P. sojae* isolates is critical for the management of PRSR using the selected *Rps* genes [13,19,22,24,25,26]. The pathotypes of *P. sojae* were determined based on the resistance or susceptibility of the differentials carrying only a single *Rps* allele after inoculation of each isolate using the hypocotyl inoculation technique [27]. The hypocotyl inoculation assays demonstrated whether each *Rps* gene can effectively protect the soybean plants against the tested *P. sojae* isolates [14,27]. These studies demonstrated that the pathotype diversity of *P. sojae* varies by country and geographical region.

In addition to the phenotypic tests using the soybean differentials for *Rps* resistance, molecular techniques have been applied to estimate the extent of genetic diversity among the *P. sojae* isolates. Randomly amplified polymorphic DNA (RAPD) markers were used to detect the genetic variability among *P. sojae* isolates among the different states in the United States [28] and even among isolates from the same geographic origin in Argentina [29]. RAPD markers were also used to confirm the sexual recombination of *P. sojae*, interspecific hybrids, and segregation of avirulence genes in vitro [30,31]. Simple sequence repeat (SSR) markers were developed based on the transcriptome and genome sequence of *P. sojae* [4]. Inter-simple sequence repeat (ISSR) markers could detect abundant genetic diversity among the *P. sojae* isolates in China, and there was frequent genetic flow between different geographical sources [16]. Whole genome sequencing of 31 *P. sojae* isolates identified variations in *Avr* genes that impair soybean infection [32]. Subsequently, discriminant haplotypes for seven *Avr* genes were projected to predict pathotypes obtained from bioassays [33]. Using this molecular assay, more recently, hundreds of *P. sojae* isolates were characterized for their pathotypes, and the evolution of relative pathotype diversity was compared among the three survey periods in Canada [26].

*Phytophthora sojae* was first isolated in the Republic of Korea in the late 1990s [6]. There were no follow-up studies for two decades, but the field received much attention, as converted paddy fields are being widely used for soybean production in recent years. Kang et al. (2019) reported the phenotypic reactions of 20 Korean soybean cultivars to four *P. sojae* isolates, which revealed that the isolates differed genetically. Genetic linkage analysis identified resistance gene loci in a few Korean resistant varieties [34,35,36,37]. Yet, the pathogenicity and genetic diversity of *P. sojae* isolates remain largely unknown in the Republic of Korea. Understanding the pathogenicity and pathotypes of local *P. sojae* isolates is critical to enable efficient decision-making in pathotype-driven soybean breeding programs for Phytophthora root rot in the Republic of Korea [25]. Thus, the objectives of the present study were to (i) investigate the pathotypes of six *P. sojae* isolates collected in the Republic of Korea based on phenotypic assays and (ii) analyze their genetic diversity using molecular markers.

## 2. Methods

### 2.1. Isolates of Phytophthora sojae

**Figure 1:**
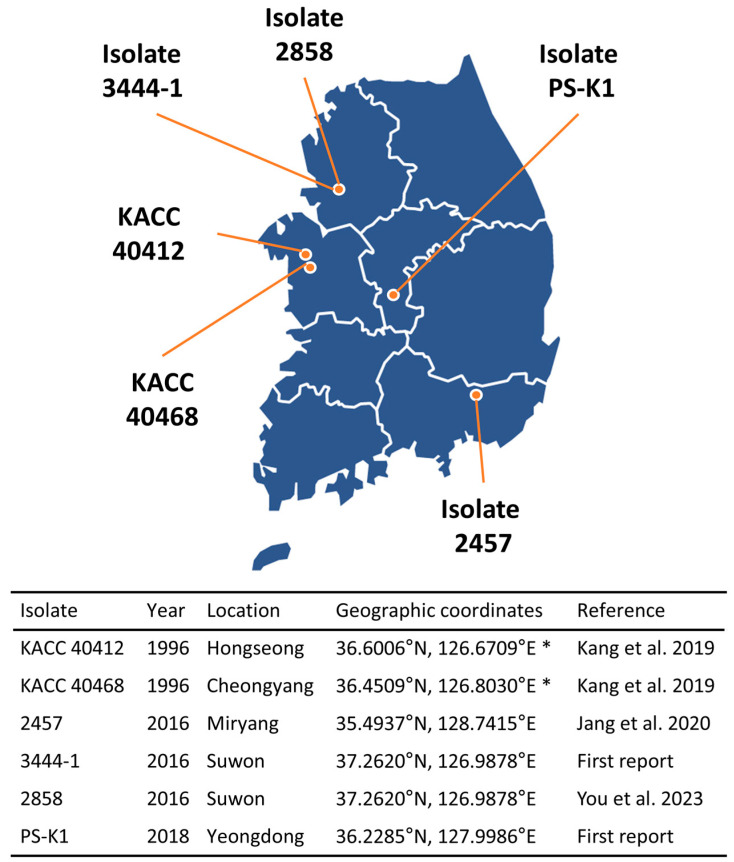
The years and geographic regions of isolation of the six *Phytophthora sojae* isolates. Note that for two old isolates, KACC 40412 and 40468, the actual geographic coordinates (indicated by asterisks) are not available, so approximate coordinates of the corresponding cities are given [34,35,38].

Six isolates of *P. sojae* were investigated for their pathotypes and genetic diversity (Figure 1). Though the small number of isolates was not expected to comprehensively embrace high levels of genetic diversity, only six *P. sojae* isolates were investigated due to the lack of available isolates. Two isolates, namely, KACC 40412 and 40468, were obtained from the Korean Agricultural Culture Collection (KACC), National Agrobiodiversity Center, Rural Development Administration, Wanju-gun, Jeollabuk-do, Republic of Korea. Both isolates were identified in Hongseong and Cheongyang, Chungcheongnam-do, Republic of Korea, in 1996. Three isolates, 2457, 3444-1, and 2858, collected from soybean fields in 2016, were obtained from the two departments of National Institute of Crop Science, Republic of Korea. Isolate 2457 was isolated in Miryang, Gyeongsangnam-do, and isolates 3444-1 and 2858 in Suwon, Gyeonggi-do, Republic of Korea, respectively, in 2016. Isolate PS-K1 was discovered from a soybean field in Youngdong-gun, Chungcheongbuk-do, Republic of Korea, in 2018. Two isolates of *P. infestans* and *P. capsici* were used as negative controls for the genotypic assay. The former was obtained from the Highland Agriculture Research Institute, Pyeongchang-gun, Gangwon-do, Republic of Korea, and the latter from the Gyeongbuk Agricultural Research and Extension Service, Gyeongsangbuk-do, Republic of Korea.

### 2.2. Pathotype Characterization

For the phenotypic assay, the hypocotyl inoculation technique was used to evaluate the pathotypes of the six isolates against 15 differentials for resistance against *P. sojae*, including one genotype with a susceptible allele (*rps*) and 14 with a single known *Rps* allele [27]. In brief, 12–15 seedlings of each differential were grown in a 13 cm-diameter plastic pot for seven days. Three 5 × 5 mm *P. sojae*-colonized agar plugs were placed on V8 agar media, and *P. sojae* mycelia grew on V8 agar media in the controlled incubator (25 °C, dark) for 7 days. *P. sojae*-grown V8 agar media were macerated by passing through a 50 mL syringe twice and then transferred into a 10 mL syringe with an 18-gauge needle. A 1 cm slit was made 1 cm below the cotyledon on the hypocotyl of seedlings by the needle tip. Approximately 0.2 to 0.4 mL of mycelial slurry was injected into the slit. Inoculated seedlings were placed in a growth chamber in the dark overnight at 25 °C with >80% moisture, then incubated in a 14 h/10 h (day/night) condition at 25 °C. Typically, susceptible plants died seven days after hypocotyl inoculation, whereas resistant plants survived by developing a hypersensitivity response. The reaction of each differential was determined by the percentage of seedlings alive or dead seven days after inoculation according to the following criteria: resistant if <20% of seedlings were dead, intermediate if 20 to 80% of seedlings were dead, and susceptible if >80% of plants were dead. The experiment was replicated at least three times.

### 2.3. DNA Extraction and Simple Sequence Repeat (SSR) Marker Genotyping

For genotypic assays, the six isolates of *P. sojae* and the two negative controls were grown on 10% V8 juice agar media in dark conditions at 25 °C for 10 days until the mycelia fully covered the media. The DNA of each isolate was extracted from the collected mycelia using the DNeasy Plant Mini Kit (Qiagen, Hilden, Germany) according to the manufacturer’s instructions. DNA concentration was measured using a Nano-MD PDA UV-Vis Bio Spectrometer (Scinco, Seoul, Republic of Korea). The concentration was normalized to 5 ng/µL, and the DNA was stored at −20 °C until use.

Twenty-one pairs of SSR primers from Dorrance and Grünwald (2009) [4] were used to genotype the isolates of *Phytophthora* species. Polymerase chain reaction (PCR) was conducted in a SimpliAmp™ thermal cycler (Thermo Fisher Scientific, Waltham, MA, USA) with a final volume of 25 μL, including 25 ng of genomic DNA, 0.5 μL of 10 μM forward primer, 0.5 μL of 10 μM reverse primer, 0.5 μL of *Taq* polymerase (SmartGene, Daejeon, Republic of Korea), 2 μL of 2.5 mM dNTP, and 2.5 μL of 10X buffer. The PCR protocol was as follows: initial denaturation at 94 °C for 5 min; 35 cycles of denaturing at 94 °C for 30 s; annealing at 58 to 66 °C for 30 s; extension at 72 °C for 30 s; and final extension at 72 °C for 5 min. Amplified PCR products were first resolved on 4% agarose gels with SmartGene DNA-staining Bandi (SmartGene, Daejeon, Republic of Korea). After electrophoresis, the gels were visualized using UV light. Capillary electrophoresis was used to measure PCR amplicons using QIAxcel (Qiagen). Among the 21 loci, 3 were removed because of ambiguous (PS17 and PS30) or non-polymorphic (PS29) bands (Table S2 and Figure S1).

### 2.4. Analysis of Genetic Diversity

The genotypes of the 18 polymorphic SSR loci were subsequently used for analyzing genetic diversity among the isolates. Statistical parameters, including numbers of alleles per locus, major allele frequency (MAF), gene diversity, heterozygosity, and polymorphic information content (PIC), were calculated using the program PowerMarker v3.25 [39]. Among them, the number of alleles per locus and PIC are often used together or in combination with other measures of genetic diversity to provide valuable insights into the genetic diversity of isolates or the utility of genetic markers [40,41]. Principal component analysis (PCA) was conducted by using the genotypes of 18 SSR markers in R [42]. The genetic distance matrix was calculated in PowerMarker using the Nei coefficient and the bootstrap resampling process (*n* = 1000). A phylogenetic neighbor-joining (NJ) tree was then constructed in PowerMarker and inspected in MEGA 6.0 using 1000 bootstrap replicates [43].

## 3. Results and Discussion

### 3.1. Pathotypes of P. sojae Isolates in the Republic of Korea

The six *P. sojae* isolates that had been isolated in different geographical locations in the Republic of Korea at different years were investigated in the present study (Figure 1; see Section 2.1). The virulence pathotypes were highly variable among the six isolates based on either resistance or susceptible reaction in the 15 differentials after hypocotyl inoculations (see Section 2.2). Each isolate showed virulence interaction with 8–15 differentials (Table 1). A virulence interaction indicates that the differential is not resistant to the specific isolate tested, suggesting that the corresponding known *Rps* allele fails to defend against isolates with such pathotypes. The pathotype complexities of the isolates PS-K1 and 40412, defined as the number of *Rps* genes with a susceptible response [14], were found to be high. The isolate PS-K1 showed virulence interactions with all 15 differentials (vir 1a, 1b, 1c, 1d, 1k, 2, 3a, 3b, 3c, 4, 5, 6, 7, and 8), and the isolate 40412 displayed virulence interactions with 12 differentials (vir 1a, 1b, 1c, 1d, 1k, 3a, 3b, 3c, 4, 5, 7, and 8) (Table 1). Two other isolates also had high levels of pathogenicity. The isolate 40468 (vir 1a, 1b, 1d, 3a, 3b, 3c, 4, 5, 6, 7, and 8) exhibited 12 compatible interactions with 1 intermediate reaction in *Rps*2, and the isolate 2457 (vir 1a, 1b, 2, 3a, 3b, 3c, 4, 5, 6, and 7) showed compatible interactions with 11 differentials. The pathotype complexities of the isolates 2858 and 3444-1 were found to be relatively lower than those of the aforementioned isolates. It was determined to be 10 for isolate 2858, comprising vir 1a, 1b, 3a, 3b, 3c, 5, 6, 7, and 8 (Table 1), while isolate 3444–1 exhibited a pathotype complexity of 8, comprising vir 1a, 1c, 1k, 2, 3a, 5, and 8.

The present study focused on a limited number of isolates, which were all available in the Republic of Korea, though previous studies conducted in other countries have typically analyzed tens to hundreds of *P. sojae* isolates to elucidate pathotype diversity, geographical distribution/complexity, and temporal shifts [12,13,14,17,20,26]. Notably, the results of this study revealed considerably high pathotype complexity ranging from 9 to 15 among Korean *P. sojae* isolates (Table 1), which surpass those documented in other nations, with reported ranges of 1 to 7, 1 to 12, and 3 to 10 in the Canadian, USA, and Brazilian isolates, respectively [13,14,26]. A recent study reported a mean pathotype complexity of 6.7 from over 400 isolates discovered in the four North Central states of the USA [44]. According to time-series tracking, *P. sojae* pathotype complexity and virulence have eventually increased in several countries, including Brazil [13], Argentina [45,46], Ontario and Manitoba, Canada [47,48], and the Jilin and Heilongjiang region of China [49,50]. 

The observed frequencies of virulence to each *Rps* allele of the six *P. sojae* isolates differed from those documented in other countries. For instance, the differentials carrying *Rps*1a, 3a, or 5 exhibited susceptibilities to all six isolates, whereas those carrying *Rps*1c, 1d, and 1k alleles were susceptible to half of the six isolates. In Japan, 90% and 33% of *P. sojae* isolates collected from three regions displayed virulence against differentials carrying *Rps*6 and *Rps*1a+7, respectively, whereas differentials containing *Rps*1d were not susceptible to over 50 isolates [51]. In Jilin, China, >57% of the isolates were virulent against *Rps*1a, 1b, 1d, 6, and 7 [50]. Similarly, in Brazil, differentials for *Rps*1d and *Rps*7 exhibited susceptibility to 37 tested isolates, while *Rps*2, 3c, 4, and 6 differentials displayed susceptibility to 70–89% of the tested isolates [13]. Moreover, in Canada, differentials containing *Rps*1a, 1c, and 1d were susceptible to 53–87% of the 295 isolates [26]. In the USA, pathotypes have been extensively investigated, with their numbers ranging from 2 to 144 per state [14,18]. The prevalent utilization of a limited number of *Rps* alleles in disease management constitutes a crucial factor contributing to the divergence in pathotypes [8,14]. Overall, it could be concluded that the six isolates used in the present study exhibited different virulence pathotypes, a degree of existing genetic diversity, and discriminability from those reported in other countries, although the considerable range of genetic diversity was unexpected, which is typically observed from hundreds of isolates.

### 3.2. Management of Phytophthora Root Rot with Respect to the Pathotypes of P. sojae

The management of PRSR has mainly depended on the cultivation of resistant soybean varieties that have been developed by introgressions of one or more *Rps* genes (Dorrance 2018). Genetic diversity within *P. sojae* isolates affects the efficacy of resistance genes in soybean cultivars and the strategies employed for disease management [52,53]. The efficacy of resistance genes (*Rps* genes) may be compromised by the emergence of new pathotypes of *P. sojae* that are capable of overcoming specific resistances. The genetic diversity of the pathogen isolates indicates that a combination of resistance genes may be necessary to provide comprehensive and long-lasting protection against the disease. To forestall the breakdown of resistance, it may be advantageous to implement a strategy of rotation or pyramiding (the combination of multiple resistance genes in a single cultivar) within the context of soybean breeding programs. This strategy is contingent upon a comprehensive understanding of the genetic diversity and virulence profiles of the local *P. sojae* isolates [52]. The potential for rapid evolution in *P. sojae* presents a significant challenge to the maintenance of the effectiveness of resistance genes. This rapid adaptability necessitates continuous research and development efforts to identify new resistance sources and to comprehend the evolving pathogen isolates. A comprehensive understanding of plant–pathogen interactions is essential for the development of novel pathogen-informed breeding strategies that transcend the limitations of traditional breeding approaches [25].

The data from our study indicates that all six isolates were virulent to *Rps*1a, 3a, and 5. This suggests that the cognate *Rps* alleles may offer limited efficacy against the *P. sojae* isolates prevalent in the Republic of Korea. However, considering the distribution of resistance reactions, *Rps*1c, 1d, and 1k demonstrate the potential for effectively conferring protection against such pathotypes of *P. sojae* isolates. Therefore, these resistant alleles should be prioritized in future breeding initiatives for deployment in susceptible soybean cultivars. In contrast, none of the 14 tested *Rps* alleles demonstrated resistance against infection by the isolates PS-K1 and KACC 40412. This finding underscores the necessity for novel *Rps* alleles sourced from diverse genetic reservoirs to enhance the management efficacy. This result aligns with prior research, wherein none of the evaluated soybean varieties exhibited resistance against KACC 40412, with only a few demonstrating intermediate resistance [38].

### 3.3. Genetic Diversity Assessed via SSR Marker Genotyping

In order to ascertain the extent of genetic diversity present among *P. sojae* isolates, several genetic parameters were calculated from SSR genotypic data. With regard to allelic richness, two to five alleles were identified per locus, resulting in a total of 68 alleles and a mean of 3.8 (Table 2). These findings are consistent with the 2.5 alleles per locus and heterozygosity of 0.015 observed in a large number of *P. sojae* isolates in Ohio, USA [4]. The observed heterozygosity was 0.0 for 17 of the 18 loci, with an overall mean of 0.019 (Table 2). The heterozygosity was not high and agreed with the homothallic nature of *P. sojae* but also indicated that rare chances of outcrossing also exist, as evidenced by previous reports [31,54]. The major allele frequency (MAF) ranged between 0.333 and 0.833, with a mean of 0.481. Of the 18 loci, 10 displayed a MAF of 0.333, while only 1 locus showed a MAF of 0.833. Along with the mean allele number per locus, this MAF frequency distribution demonstrated a high level of allelic variation among the six isolates at the nucleotide level. The gene diversity was defined as the probability that two randomly chosen alleles from each isolate are different; thus, it generally decreased when the MAF was higher (Table 2). The mean genetic diversity was 0.624 (Table 2), and the SSR markers were effective in elucidating the genetic diversity of each locus among the six isolates. The mean PIC was 0.579, with four SSR loci exhibiting high informativity (PIC > 0.7) and only one SSR locus having a PIC < 0.25 (Table 2). The number of alleles that can be characterized by SSR markers is positively correlated with the PIC values. This was demonstrated in the present study, where significant positive correlations were found among the number of alleles per locus, genetic diversity, and PIC values (Pearson’s correlation, *r* > 0.9, *p* < 0.001) (Table 2).

The number of alleles per locus serves as a basic indicator of genetic variation. Higher allelic richness signifies greater genetic diversity, and tracking the changes in allelic richness over time facilitates the assessment of the impact of various factors, such as habitat loss, isolate fragmentation, or selective pressures on genetic diversity [55]. Additionally, PIC serves as a measure of the informativeness of a genetic marker, such as a microsatellite or SNP [56]. It quantifies the level of polymorphism within a marker by considering both the frequency of different alleles and their relative frequencies and, thus, is commonly used in studying the genetic structures of the populations of a species to select the most informative molecular markers to differentiate sub-populations [57]. Furthermore, it estimates the capacity to detect polymorphisms among individuals, typically categorized as slight, moderate, or high, based on the values <0.25, 0.25–0.5, or >0.5, respectively [58]. Similar to the phenotypic results, the limited sample size might preclude the assessment of a sufficiently broad range of variation to match the anticipated range expected from hundreds of isolates. However, an evaluation of the genotypic assay results indicated that the six isolates exhibited a high degree of genetic variations.

**Figure 2:**
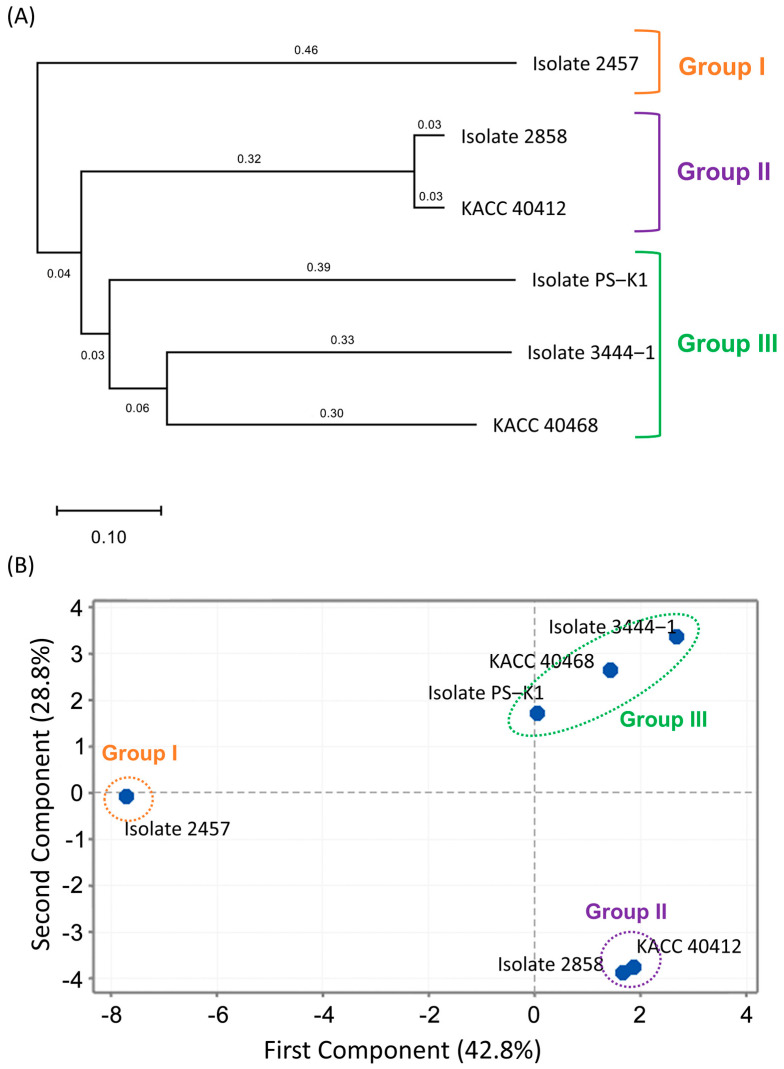
Genetic dissimilarity among six isolates of *Phytophthora sojae* based on 18 simple sequence repeat (SSR) marker genotypes. (**A**) Phylogenetic tree, (**B**) Principal component analysis.

A phylogenetic analysis was conducted using the SSR genotypes of the 18 loci to delineate the relationships among the isolates. This analysis revealed that the isolate could be segregated into three groups: I, II, and III (Figure 2A). The isolate 2457 (Group I) exhibited distinctive characteristics compared to the other five isolates. Within Group II, KACC 40412 and isolate 2858 exhibited a closer genetic affinity, indicating a substantial degree of genetic similarity. The remaining three isolates were assigned to Group III, although they exhibited distinct characteristics from each other. Similarly, principal component analysis (PCA) was employed to examine the multidimensional relatedness among the six isolates. The first and second principal components accounted for 42.8% and 28.8% of the total variation, respectively (Figure 2B). It is noteworthy that the PCA scatter plot and the phylogenetic tree exhibited concordant patterns. On analyzing where and when the *P. sojae* isolates were isolated, it was difficult to find any trend based on the geography and time of year (Figure 1 and Figure 2).

### 3.4. Considerations in Applying the Molecular Technique in Assessing the Genetic Diversity of P. sojae

Randomly amplified polymorphic DNA (RAPD), simple sequence repeat (SSR), and inter-simple sequence repeat (ISSR) markers have been used to detect genetic variability in *P. sojae* populations in several countries [4,16,28]. These molecular approaches can be used to confirm sexual recombination, segregation of avirulence genes, and gene flow in *P. sojae* [15,16,29,31]. In the present study, phylogenetic analysis and PCA based on SSR genotypes successfully differentiated the six isolates, but the molecular results were different from the phenotypic results. For example, KACC 40412 and the isolate 2858 demonstrated high genetic similarity according to SSR genotypes; the Nei’s genetic distance between the two isolates was approximately 0.05, whereas the distance for all other pairs was over 0.6 (Figure 2 and Table S1). However, their pathotypes were distinguishable; *Rps 1c*, *1d*, *1k*, *2*, and *4* can provide protection against the isolate 2858 (Table 1). KACC 40412 and the isolate 2858 are the two isolates with the first and second highest pathotype complexity but were separately grouped (Figure 2). Similar results were also reported in a previous study conducted using USA isolates; grouping by SSR genotypes and phenotypic response differed when much higher numbers of *P. sojae* isolates were used [8,18].

Basically, the two types of data are always expected to match each other. A possible explanation for these results is as follows. These SSR motifs were initially discovered from the transcript sequences and draft genome sequences of some *Phythophthora* spp., and those with polymorphism among the 38 *P. sojae* isolates were selected from preliminary tests [4]. Since there was no consideration of association with virulence or avirulence during the process of the SSR marker development, the selected SSR markers are neither necessarily closely linked nor located in the vicinity of *Avr* genes. Such random genomic positions can generally help estimate the genetic diversity among isolates but also have limitations while determining the association between the SSR genotypes and phenotypic results. In addition, even though an SSR motif and *Avr* are closely linked, the evolutionary history of *Avr* genes and the SSR motif may differ during the evolutionary process. *P. sojae* is generally known as homothallic, though evidence of rare outcrossing and gene flow between isolates was reported in a few studies [31,44,54]. The random nucleotide mutation and changes in the repeat number of the SSR motif can naturally and independently occur within a lineage over generations. The selection pressure on *Avr* genes also affected the frequency distribution of each allelic variation and the interaction between the two organisms; therefore, genetic similarities based on SSR genotypes might not match the phenotypic results between isolates.

To overcome this drawback of SSR markers, recent investigations using high-throughput sequencing technology provide an alternative solution to the weak or no association between phenotypic responses and SSR marker genotypes. Whole-genome sequencing was employed to analyze 31 *P. sojae* isolates, uncovering nucleotide sequence variations within *Avr* genes associated with the diminished soybean infectivity [32]. Subsequent research used discriminant haplotypes pertaining to the seven *Avr* genes to forecast pathotypes discerned through phenotypic assays, where molecular prediction was equivalent to the pathotypes determined by phenotypic assays [33]. Then, approximately 100 *P. sojae* isolates were characterized for their pathotypes according to single nucleotide polymorphism (SNP), and the evolution of relative pathotype diversity was compared among the three survey periods in Canada [26]. This SNP-based molecular approach has shown improved accuracy in predicting phenotypic assay results [26]. Despite the widespread use of phenotypic assays relying on the reactions of differential genotypes, they have limitations in that they are labor-intensive, can yield intermediate reactions, produce false positives/negatives, and bypass the natural method of zoospore infection of the root system [26].

In conclusion, this study demonstrated that the six isolates are genetically diverse based on molecular markers and have differing virulence of pathotypes based on phenotypic assays. Given the relatively small sample size of six *P. sojae* isolates included in the present genetic study, the interpretation and inferences derived from this investigation may be constrained and divergent from those that could be obtained from a larger array of *P. sojae* isolates in future analyses. Nonetheless, it is hypothesized that these preliminary results, though limited in scope, will serve as a foundation for elucidating the virulence pathotypes of the extant *P. sojae* isolates present within the soybean fields in the Republic of Korea. Generally, population genetic analysis is a potent instrument for comprehending the emergence and adaptation of pathogens. Nevertheless, ascertaining the genetic composition of populations necessitates a sophisticated understanding of the inherent characteristics of pathogens, in addition to the meticulous collection of a substantial number of samples through a judicious approach [59]. From this standpoint, it is imperative that a more extensive array of *P. sojae* isolates is amassed for the purpose of enhancing the comprehension of the geographical distribution and frequency of virulence pathotypes of *P. sojae* in the Republic of Korea in the forthcoming years. The utilization of nationwide, time-series data will facilitate the realization of two primary objectives: first, tracing the pathotype complexity in the entire population or sub-population(s) across different provinces in the Republic of Korea, and second, the selection of specific *Rps* genes that may prove more effective in specific locations for pathotype-driven breeding. This would also allow for the identification of temporal and spatial changes in the diversity of Korean *P. sojae* isolates. After a large-scale investigation is conducted, virulence pathotypes of the predominant isolates will be key for selecting the preferred *Rps* sources when developing *P. sojae*-resistant soybean varieties in breeding programs.

## Figures and Tables

**Table 1:** Reactions of fifteen *Rps* differentials against six isolates of *Phytophthora sojae* from the Republic of Korea.

Differentials	*Rps* Allele	Responses by Isolate ^a^						% Resistance Response
		KACC 40412	KACC 40468	Isolate 2457	Isolate 3444-1	Isolate 2858	Isolate PS-K1	
Williams	*rps*	S	S	S	S	S	S	0
Harlon	*Rps*1a	S	S	S	S	S	S	0
Harosoy 13XX	*Rps*1b	S	S	S	R	S	S	17
Williams 79	*Rps*1c	S	R	R	S	R	S	50
PI 103091	*Rps*1d	S	S	R	R	R	S	50
Williams 82	*Rps*1k	S	R	R	S	R	S	50
L76-1988	*Rps*2	I	I	S	S	R	S	17
L83-570	*Rps*3a	S	S	S	S	S	S	0
PRX 146-36	*Rps*3b	S	S	S	R	S	S	17
PRX 145-48	*Rps*3c	S	S	S	R	S	S	17
L85-2352	*Rps*4	S	S	S	R	R	S	33
L85-3059	*Rps*5	S	S	S	S	S	S	0
Harosoy 62XX	*Rps*6	I	S	S	R	S	S	17
Harosoy	*Rps*7	S	S	S	R	S	S	17
PI 399073	*Rps*8	S	S	R	S	S	S	17
Pathotype complexity		13	12	11	8	10	15	

^a^ R, S, and I denote resistant, susceptible, and intermediate reactions, respectively.

**Table 2:** Parameter estimates for genetic diversity of six isolates of *Phytophthora sojae* based on eighteen simple sequence repeat (SSR) markers.

SSR Marker	Major Allele Frequency	Number of Allele	Gene Diversity	Heterozygosity Observed	Polymorphic Information Content (PIC)
PS01	0.333	5	0.778	0.000	0.744
PS04	0.500	4	0.667	0.000	0.620
PS05	0.333	4	0.722	0.000	0.671
PS06	0.333	4	0.722	0.000	0.671
PS07	0.833	3	0.292	0.333	0.272
PS10	0.500	4	0.667	0.000	0.620
PS12	0.333	4	0.722	0.000	0.671
PS16	0.667	3	0.500	0.000	0.449
PS18	0.500	3	0.611	0.000	0.535
PS19	0.333	4	0.722	0.000	0.671
PS20	0.333	4	0.722	0.000	0.671
PS24	0.333	4	0.722	0.000	0.671
PS25	0.833	2	0.278	0.000	0.239
PS27	0.333	5	0.778	0.000	0.744
PS30	0.333	5	0.778	0.000	0.744
PS33	0.333	5	0.778	0.000	0.744
PS36	0.833	2	0.278	0.000	0.239
PS38	0.667	3	0.500	0.000	0.449
Mean	0.481	3.8	0.624	0.019	0.579

## Data Availability

The data sets used and/or analyzed during the current study are available from the corresponding author on reasonable request.

## Supplementary Materials

The following supporting information can be downloaded at https://www.mdpi.com/article/10.3390/microorganisms13030478/s1: Table S1. Matrix of genetic distance calculated among six isolates of *Phytophthora sojae.* Table S2. Variation in fragment sizes amplified by twenty-one simple sequence repeat (SSR) markers in six isolates of *Phytophthora sojae.* Figure S1. Examples of polymorphic fragments amplified using four simple sequence repeat (SSR) markers (PS01, PS27, PS30, and PS33). Lanes 1–6 indicate *Phytophthora sojae* KACC 40412, KACC 40468, isolate 2457, isolate 3444-1, isolate 2858, and isolate PS-K1, respectively. Lanes 7 and 8 present two negative controls, *P. capsica* and *P. infestans*.
