# Supplementary material for: Pathotypes and Simple Sequence Repeat (SSR)-Based Genetic Diversity of Phytophthora sojae Isolates in the Republic of Korea
Source: Microorganisms. 2025 Feb 21;13(3):478. doi: 10.3390/microorganisms13030478 (PMC11945044; doi:10.3390/microorganisms13030478)
Supplement: Supplementary file 1 [file microorganisms-13-00478-s001.zip › Supple Tables.pdf]

Table S1. Matrix of genetic distance calculated among six isolates of *Phytophthora sojae*

Isolates	Isolate 2457	Isolate 2858	Isolate 3444-1	Isolate PS-K1	KACC 40412	KACC 40468
Isolate 2457		0.8496	0.9722	0.9052	0.8496	0.8496
Isolate 2858			0.7385	0.7778	0.0556	0.7222
Isolate 3444-1				0.7385	0.7385	0.6274
Isolate PS-K1					0.7778	0.7778
KACC. 40412						0.7222
KACC. 40468						

Table S2. Variation in fragment sizes amplified by twenty-one simple sequence repeat (SSR) markers in six isolates of *Phytophthora sojae*

SSR ID	Expected size (bp)[a]	Size (bp) of race2[a]	Size (bp) of amplified bands of the six isolates[b]						No. of alleles
			KACC 40412	KACC 40468	Isolate 2457	Isolate 3444-1	Isolate 2858	Isolate PS-K1	
PS01	200-450	419	354	322	296	329	354	384	5
PS04	250-320	307	277	262	260	277	277	265	4
PS05	200-300	263	223	353	312	353	223	242	4
PS06	160-200	214	202	211	205	211	202	227	4
PS07	220-240	200 / 234	214 / -	214 / -	214 / 234	214 / 222	214 / -	214 / -	3
PS10	150-250	228	173	168	187	173	173	183	4
PS12	220-306	306	288	293	311	299	288	299	4
PS17	180-230	213	-	-	-	-	-	-	0
PS16	400-500	469	406	406	385	398	406	406	3
PS18	170-200	185	194	199	194	197	194	197	3
PS19	225-275	254	269	254	254	246	269	251	4
PS20	150-225	184	161	203	210	203	161	253	4
PS24	220-260	252	275	258	252	278	275	278	4
PS25	350-400	366	367	367	367	374	367	367	2
PS27	275-325	287	326	299	329	315	326	304	5
PS29	250-280	273	253	253	253	253	253	253	1
PS30	270-300	300	327	339	281	339	317	343	5
PS33	240-267	267	271	284	260	253	271	274	5
PS36	150-220	209	221	221	218	221	221	221	2
PS37	200-370	204	-	-	-	-	-	-	0
PS38	245-270	245	252	252	270	252	252	241	3

[a] The information was referred from Dorrance and Grünwald (2009).
[b] The hyphen (-) indicates that there is no amplified band.
