# Supplementary figures and images for: Pathotypes and Simple Sequence Repeat (SSR)-Based Genetic Diversity of Phytophthora sojae Isolates in the Republic of Korea
Source: Microorganisms. 2025 Feb 21;13(3):478. doi: 10.3390/microorganisms13030478 (PMC11945044; doi:10.3390/microorganisms13030478)

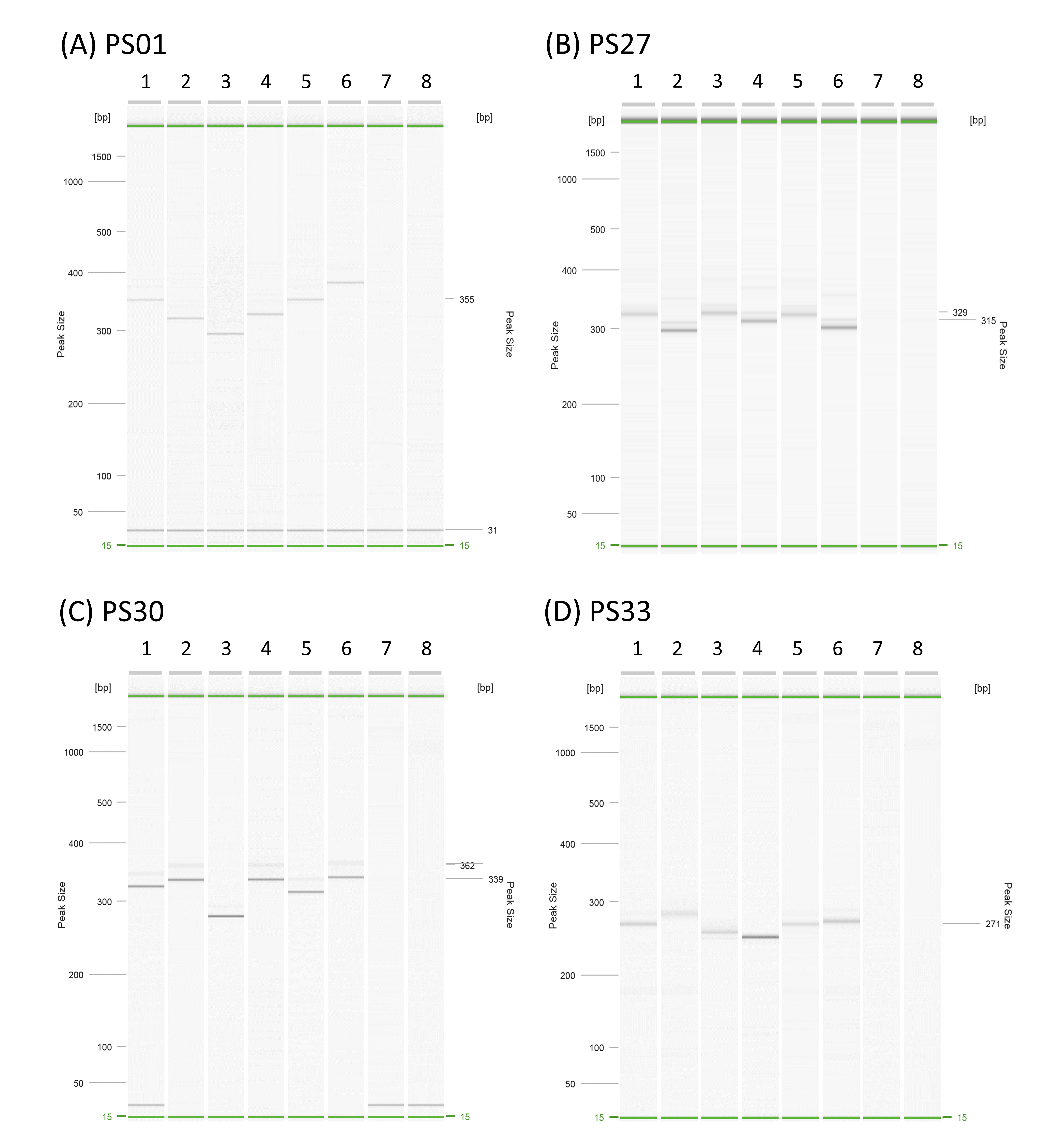

Supplement: Supplementary file 1 [file microorganisms-13-00478-s001.zip › Fig S1.TIF]
